# From Fly on the Wall to Future Colleagues: Best Practice Recommendations for Medical Student Shadowing Programs

**DOI:** 10.1002/aet2.70214

**Published:** 2026-06-23

**Authors:** Molly Estes, Danielle Matonis, McKenna Knych, Brian Barbas, Ronnie Ren, William Dixon, Michael Pasirstein, Xiao Chi Zhang, Kaitlin Bowers, Ravi R. Chauhan, Laryssa A. Patti

**Affiliations:** ^1^ Northwestern University Chicago Illinois USA; ^2^ Department of Emergency Medicine University of California Irvine Orange California USA; ^3^ Medical College of Wisconsin Milwaukee Wisconsin USA; ^4^ Loyola University Chicago—Stritch School of Medicine Maywood Illinois USA; ^5^ University of Florida Gainesville Florida USA; ^6^ Stanford University Palo Alto California USA; ^7^ Thomas Jefferson University Philadelphia Pennsylvania USA; ^8^ Campbell University Lillington North Carolina USA; ^9^ Ochsner Medical Center New Orleans Louisiana USA; ^10^ Rutgers Health Robert Wood Johnson Medical School New Brunswick New Jersey USA

## Abstract

**Background:**

Physician shadowing is a common component of early medical education and frequently represents students' first exposure to clinical practice. Despite its prevalence, existing guidance primarily targets learners rather than physicians supervising shadowing experiences. This gap is especially relevant in the emergency department (ED), where high patient acuity, workflow demands, and complex clinical environments create challenges for providing safe and meaningful observational learning. We sought to develop consensus‐based best practice recommendations for ED faculty supervising medical student shadowing.

**Methods:**

We conducted a modified Delphi study using an online survey platform to achieve panelist consensus. Ten emergency medicine educators with a mean of 7 years of experience in medical student advising and clerkship leadership participated. Panelists initially generated 70 proposed best practice recommendations, which were refined to 54 unique items after removal of duplicates. Four Delphi rounds were completed, during which participants iteratively selected and refined recommendations through anonymous voting and feedback. Items were progressively eliminated or merged according to consensus thresholds until a final set of recommendations was established.

**Results:**

Consensus was achieved on 10 best practice recommendations after four Delphi rounds. Key themes included establishing structured scheduling and orientation processes, clearly communicating expectations and eligibility requirements, limiting concurrent learners, assessing students' backgrounds and goals, assigning a designated preceptor, enforcing restrictions on direct patient care, integrating students into the clinical team, providing opportunities for questions and reflection, maintaining engagement during workflow lulls, and modeling professionalism. Panelists emphasized balancing educational value with patient safety and clinical efficiency.

**Conclusions:**

These consensus‐driven recommendations provide practical guidance for supervising medical student shadowing in the ED. Standardizing shadowing practices may transform shadowing into a structured early clinical learning experience, improving learner engagement, supporting safe educational environments, and enhancing early exposure to emergency medicine. Future research should evaluate implementation outcomes and applicability across specialties.

## 1 Introduction

Shadowing is a common component of preclinical and early clinical medical education [1, 2, 3]. During the early phases of medical school, shadowing provides students with opportunities to observe patient care, explore different specialties, and learn about various parts of the healthcare system. These experiences may play an important role in developing students' early career interests and shaping their professional identity.

Despite the prevalence and educational importance of physician shadowing, published guidance has been largely student‐focused [4, 5]. There is little evidence‐based guidance for physicians regarding how to best supervise shadowing students [6]. Current guidance focuses on precepting learners such as residents or clinical year medical students as these levels of training are cleared for direct patient interaction such as obtaining histories with collateral information or performing physical exams [7]. This is a critical gap, as early clinical or pre‐clinical medical students are a distinct group with limited ability to directly engage in patient care. Further, shadowing students may have widely varied backgrounds, prior exposures, and goals, making it challenging to consistently foster safe, high‐value learning environments for all.

Additionally, the emergency department (ED) provides a unique experience for shadowing as it offers a dynamic, high‐acuity environment where students can observe direct patient care, interprofessional teamwork, and the intersection of multiple specialties. Prior work has shown that early clinical experiences, including shadowing, can influence students' specialty choices [8, 9, 10]. This influence may be especially significant in emergency medicine (EM), as many medical schools do not require an EM clerkship or delay it to the fourth year, when many students have already selected a specialty [11]. As a result, high‐quality shadowing experiences in the ED may serve as an important early exposure to EM and function as a recruitment path to the specialty [12, 13, 14, 15]. For students who ultimately pursue other fields, shadowing in the ED may represent their first or most in‐depth interaction with EM, and the quality of this experience may shape their perceptions of EM as they pursue other specialties.

While it is established that ED shadowing is a valuable educational experience for early clinical learners, it presents many challenges for the supervising faculty involved, due to patient acuity, space limitations, workflow pressures, and the complex and often intense social situations encountered in the ED. Faculty often precept shadowing students without a structured approach or formal training while managing other teaching responsibilities. Without clear guidance or a structured approach, precepting can become burdensome, be inconsistently educational, and not always conducive to a safe learning environment [3, 6]. In order to fill the gap of consensus‐based guidance for ED faculty, this study aims to use the modified Delphi method to develop consensus‐driven best practices for ED preceptors supervising shadowing students.

## 2 Methods

### 2.1 Study Design

A modified Delphi method was performed via an online survey platform (Google Forms) to achieve consensus on best practice recommendations for medical student shadowing programs. The modified Delphi method was chosen as a methodology to achieve group consensus while avoiding the potential effects of bias and allowing for anonymous responses and feedback after each round of data analysis [16, 17, 18].

### 2.2 Setting and Participants

A group of 10 panelists was recruited for participation in this study through their association with the Council of Residency Directors Advising Students Committee in Emergency Medicine via an open recruitment call to all membership. All participants had experience in direct medical student advising, and all were either current or former Clerkship Directors. Their years of experience in student advising ranged from 3 to 11, with a mean of 7 years. The panelists were drawn from across the nation, with over half of the Society for Academic Emergency Medicine regions being represented [19], and represented a variety of practice environments, including academic, community, and hybrid settings. In addition to the 10 panelists, the study group also included the lead author, who abstained from participation in the Delphi rounds and was solely responsible for collating and analyzing the survey responses for the purpose of generating the next Delphi round (see Table 1).

**TABLE 1:** Demographic make‐up of study participants.

Panelist demographics	
Gender	
Male	54.5%
Female	45.5%
Age (years)	
35–40	54.6%
41–45	18.2%
46–50	9.1%
51–55	9.1%
Academic rank	
Assistant Professor	63.6%
Associate Professor	36.4%
Years in practice	
1–5	9.1%
6–10	63.6%
11–15	9.1%
> 15	18.2%
Years in student clerkship leadership	
1–5	36.4%
6–10	63.6%
Years as a student advisor	
1–5	18.2%
6–10	63.6%
11–15	18.2%
Primary institution practice site	
Academic	90.9%
Community	9.1%
County	0.0%
Urban	45.5%
Suburban	54.5%
Rural	0.0%
SAEM geographic region	
New England	0.0%
Mid‐Atlantic	27.3%
Midwest	0.0%
Southeastern	18.2%
Great Plains	27.3%
Western	18.2%
South Central	9.1%

### 2.3 Study Protocol

The panel was asked to complete a survey to generate their personal best practice (BP) recommendations drawn from their experience in working with and teaching students. The survey prompt read, “Please provide a minimum of 5 (maximum of 10) of your best practice recommendations to preceptors who are having a medical student shadow them.” All panelists were required to submit a minimum of 5 and a maximum of 10 BP recommendations. This generated a starting list of 70 BP recommendations (see Appendix S1). The lead author analyzed the initial BP recommendations to remove duplicates and analyze if certain items had enough variation in theme, wording, or emphasis to be included in the first round. Sixteen items were initially eliminated, resulting in 54 items proceeding into the Delphi rounds.

### 2.4 Data Analysis

**FIGURE 1:**
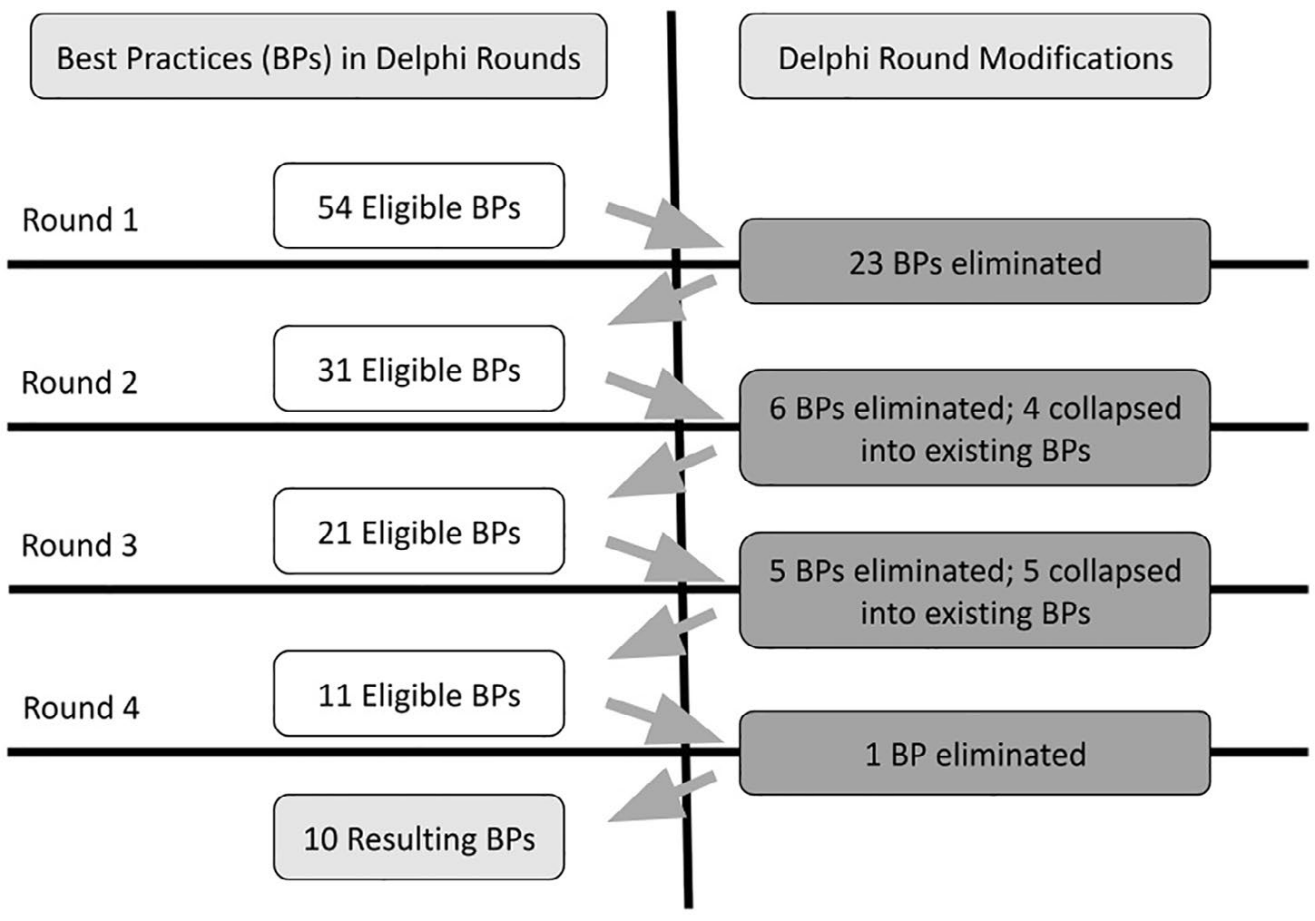
Selection process of best practice (BP) recommendations per Delphi round.

Four rounds of surveys were completed in total (see Figure 1). The response rate was 80% (8 out of 10 panelists) or higher for each round. In Delphi Round 1, participants selected 10 of the 54 items to move into the next round. A total of 23 items received no votes for inclusion in the next round and were eliminated, resulting in 31 items included for the next round. In Round 2, participants selected 10 of 31 items to move into Round 3. A total of 6 items received no votes for inclusion in the next round and were eliminated. An additional 4 items were condensed into similarly themed items, resulting in 21 items included for Round 3. In Round 3, participants selected 7 of 21 items to include in the final recommendations. A total of 5 items received no votes for inclusion in the next round and were eliminated. An additional 5 items were condensed into similarly themed items, resulting in 11 items included for the last round. In Round 4, participants selected 1 of the remaining 11 items to eliminate. Of the remaining 11 items, 5 received no votes for elimination, 3 received 1 vote for elimination, 2 received 2 votes for elimination, and only 1 item received a majority of 3 votes for elimination. This left a final tally of 10 BP recommendations. This total was chosen by panelist consensus as able to provide robust but not overwhelming guidance for future utilizers. These recommendations were further examined by the entire panel for cohesiveness and detail of wording to ensure they were representative of the entire panel's opinion and experience.

## 3 Results

After 4 Delphi rounds, there was panel consensus for the final 10 best practice recommendations. These best practices were compiled by the lead author and then submitted to the panel for further edits and descriptions (see Table 2).

**TABLE 2:** Final best practices recommendations for student shadowing in the ED.

Student shadowing best practices	
Establish a clear structure for shadowing	Use a centralized, accessible process for students to sign up, including a designated contact and clear communication of dates/times availability. Clarify eligibility (e.g., internal vs. external students) and any required paperwork or training (e.g., proof of vaccination, HIPAA training). Share expectations for communication, including if the student is expected to contact the attending in advance, and give brief orientation materials ahead of time, including arrival time, attire, meeting location, and behavior expectations
Limit the total number of students and other learners to a manageable level	When faculty are supervising multiple learners, shadowing students may have fewer opportunities to observe direct patient care, and faculty may have less time to dedicate to them. Conversely, the presence of a student may also draw faculty attention away from the learners they are actively supervising. Faculty and administrators scheduling students should be mindful of this dynamic to ensure that both students and other learners have a meaningful experience
Identify the student's healthcare background, experience level, and expectations	This should occur at the beginning of the shift or before it starts. Preceptors should inquire about the student's education level, healthcare background, and goals for this experience. Students should also be asked about their interests or any questions they might have. This will enable preceptors to tailor the shadowing experience (and its learning objectives) to the specific student
Introduce your student to the team and to patients	Start the shift by introducing your student to other members of the team—residents, nurses, staff, and any other learners. Try to make the student feel welcomed and included. Also, when seeing patients, be sure to introduce the shadower and briefly explain their role (e.g., “This is Ash, a medical student shadowing me today”)
Each student should have a designated preceptor for the shift	If interesting experiences are happening in your ED that you are not directly involved with (such as a consultant performing a procedure, a colleague running a code, a nurse placing an ultrasound‐guided IV, etc.), encourage your student to watch that experience with your colleague's agreement
Select senior residents may precept students	Senior residents must receive adequate training and program leadership approval before precepting. This should be a voluntary opportunity for interested residents only
Enforce restrictions on direct patient contact	Students are observers. They should not personally perform any patient care, such as history taking, physical exams, or invasive procedures. This is for the safety and legal protection of both patients and students, and must be enforced. Exceptions may apply to clinical students or program participants to whom your institution has granted additional privileges. These students must receive appropriate supervision from their preceptor
Create opportunities for students to ask questions on shift	Early in the shift, it is helpful to normalize that there may be busy periods when the faculty member needs to defer questions, but there will also be quieter periods during which questions and discussion are encouraged. Check in frequently after critical cases, trauma activations, or unexpected deaths, which can be intense for students. If such events occur, check in with the student afterward or plan to debrief during a quieter moment
Find opportunities for students to stay engaged during lulls	There may be times when a physician must document for prolonged periods and need to focus. Arrange alternative activities for the students, such as pairing them with one of your colleagues, senior learners, nurses, or techs, to observe different aspects of patient care
Be your (best) self	Students want to see the day‐to‐day life of emergency medicine physicians, but remember to model professionalism in language, attitudes, and behavior. Students may not have the experience or context to interpret dark humor, sarcasm, or frustration. Faculty should be mindful of tone, attitude towards patients, and interactions with team members and other consultants

## 4 Discussion

This study presents best practices from medical education panelists for emergency medicine physicians supervising shadowing medical students. By following the proposed guidelines, preceptors can ensure a more consistent experience for students that maintains educational value, encourages a safe learning environment, and promotes the specialty without adding additional burden to already busy shifts.

A major theme reflected in these guidelines is maintaining a high‐quality and safe learning environment [20], which starts by defining clear and consistent requirements and providing proper orientation [15]. A designated point person allows students to understand to whom they can definitively pose questions. An initial brief assessment of the student's prior experiences and goals for the shadowing experience, as well as introducing them to team members, can also help them feel welcome and part of the team. Since students may be exposed to intense aspects of healthcare such as agitated patients, severe injuries, and death for the first time, it is important to give them the time and space to ask questions and to debrief difficult scenarios. If their designated preceptor is pulled in a different direction, utilizing a resident teacher has been shown to augment resident education without detracting from their own experience or productivity [21, 22].

Maintaining a quality shadowing experience can also influence students' perception of the specialty. This is why we promote the guideline of “being your best self”. Many medical schools still do not require emergency medicine as a rotation, or if they do, it is not offered until the fourth year, after most students have decided on a specialty [11, 23]. These early shadowing opportunities can therefore be important exposure and help recruit interested students [12, 13, 14, 15]. Promoting the specialty of EM in a positive light is especially important in the current era of slowly recovering applicant numbers [14]. While preceptors are encouraged to provide a realistic portrayal of the ED, they should be cognizant that students do not have the same prior experience or context and may have more intense perceptions of things such as dark humor and negative comments about emergency medicine or other specialties. Positively promoting our roles in the healthcare system can help draw more students to the specialty and additionally have an impact on students who ultimately go into other specialties but will still interface with emergency medicine.

All physicians must juggle multiple demands in any given workday. Emergency physicians in particular must balance high acuity care, charting, and frequent interruptions [23, 24, 25, 26]. Adding a teaching responsibility should not significantly detract from these obligations. Limiting the number of all learners so that the preceptor is able to focus on their own clinical work while appropriately supervising and teaching each trainee [27]. Preceptors should also engage other members of the team for the student to shadow, which can not only introduce students to interdisciplinary teamwork but also provide some time for the preceptor to catch up on charting or other crucial tasks [28].

These guidelines also address some of the impacts of shadowing on patients. In medicine, prioritizing patient care must also balance the need for educational progression [29]. For example, allowing a trainee to perform a procedure so they may gain skills instead of having an attending perform all procedures. Any preceptor with shadowing students should consider patient comfort and ask permission to have students in the room when appropriate. System processes should include informing and consenting patients about the presence of students in clinical environments. This is also why we recommend having clearly defined guidelines on a student's potential direct participation in patient care, including various clinical and administrative tasks.

Future studies should investigate the widespread perception of the proposed guidelines and incorporate a broader consensus from other educators. Additionally, the effectiveness of these guidelines should be evaluated after they are put into use, and whether they are generalizable to other specialties. Also, while these recommendations focus primarily on medical students, further work should explore their application and possible adaptations for other types of learners, such as undergraduate students who commonly seek shadowing experiences while considering a career in healthcare [1].

## 5 Limitations

The study has limitations that must be considered in interpreting the results. The choice of panels may not be representative of the larger pool of EM medical student advisors. Although efforts were made to gather a broad representation of advisors, it is possible that different results may have been obtained from a panel composed of different individuals. Similarly, the use of a modified Delphi process as a study technique may have limited generalizability, as alternative study designs could have yielded different results. While the lead author remained independent of the Delphi rounds, they were solely responsible for synthesizing and merging responses between rounds rather than using a group‐based consensus process, introducing the possibility of bias [16, 17]. Some panel members did not respond to every round (although individual responses were anonymous and not tracked), and several panelists had prior collaborated through other advising‐related activities, including research and national committee work, possibly leading to preexisting agreement on the topic.

## 6 Conclusions

This study provides recommendations to guide faculty in developing meaningful pre‐clinical medical student shadowing experiences. Key elements include centralized scheduling, clear communication of expectations, structured preparation, and limiting the total number of learners to maintain educational quality. These findings support reframing shadowing from an informal observational activity into a purposeful early clinical learning experience grounded in educational best practices. Future work should focus on implementation and outcomes to evaluate how these recommendations influence student development, faculty experience, and applicability across specialties and other shadowing contexts.

## Author Contributions

**McKenna Knych:** methodology, writing – original draft, writing – review and editing. **Danielle Matonis:** methodology, writing – original draft, writing – review and editing. **Molly Estes:** conceptualization, methodology, writing – review and editing, data curation, formal analysis, investigation, writing – original draft, supervision. **Brian Barbas:** methodology, writing – original draft, writing – review and editing. **Kaitlin Bowers:** methodology, writing – original draft, writing – review and editing. **William Dixon:** methodology, writing – original draft, writing – review and editing. **Ronnie Ren:** methodology, writing – original draft, writing – review and editing. **Michael Pasirstein:** methodology, writing – original draft, writing – review and editing. **Ravi R. Chauhan:** methodology, writing – original draft, writing – review and editing. **Laryssa A. Patti:** methodology, writing – original draft, writing – review and editing, formal analysis. **Xiao Chi Zhang:** methodology, writing – original draft, writing – review and editing.

## Funding

The authors have nothing to report.

## Conflicts of Interest

The authors declare no conflicts of interest.

## Supporting information


**Appendix S1:** Original generated list of suggest best practices.

## Data Availability

The data that supports the findings of this study are available in the supporting information S1 of this article.
