# Supplementary material for: From Fly on the Wall to Future Colleagues: Best Practice Recommendations for Medical Student Shadowing Programs
Source: AEM Educ Train. 2026 Jun 23;10(3):e70214. doi: 10.1002/aet2.70214 (PMC13291185; doi:10.1002/aet2.70214)
Supplement: Supplementary file 1 — Appendix S1: Original generated list of suggest best practices. [file AET2-10-e70214-s001.docx]

Appendix 1: Original generated list of suggest best practices

| Medical students shadowing the emergency department should complete all required privacy and other training (ie HIPAA, etc) by the institution before coming the the ER. |
| --- |
| Ask students about their interests, questions, and goals and do your best to tailor the student's shadowing experience to that. |
| If your institution does not have an organized shadowing system/process for coordinating/scheduling shadowing, advocate for the creation of one. |
| Identify the goals and expectations of the shadowing student - i.e. are they here for exposure or trying to be a future EM resident? |
| Have a MINIMUM requirement of hours or sessions. |
| Have an established system with a clear owner for students to sign up - it also needs to take into consideration students from your med school versus undergrads versus CCTs from other floors, etc. |
| Model professionalism in language, attitudes, and behavior. Students may not have the experience or context to interpret dark humor, sarcasm, or frustration. Be mindful of your tone, how you speak about patients, and how you interact with the team and other physicians. |
| Allow junior students to shadow on shifts with senior learners. |
| Schedule shadowers with core faculty. |
| Medical students should be dressed appropriately with identification displayed while shadowing. |
| If they are a medical student, let them do stuff (as long as it's appropriate for their level and compliant with institutional policies). Treat them more like a preceptee than a true shadow. It gets them more engaged. |
| Offer a variety of available shift times throughout the day/week for students to shadow so they can get a full range of experiences. |
| Use a standardized framework to determine attitude in EM based on domains such as professionalism, engagement, and communication. |
| Schedule students with the same preceptor, if possible. |
| Having students come early or mid shift so that they are not hanging around for sign out or boring charting. |
| Clarify the student’s background and goals at the start of the shift. Ask why they’re shadowing—are they committed to EM, exploring it, or just seeking general clinical exposure? Also ask about their background (e.g., year in training, prior clinical experience) so you can tailor the experience and involve them at an appropriate level when possible. |
| Have an understanding of where the shadower is in their schooling. |
| Schedule students with a chief resident for a shift. |
| Shadowing students should only observe sensitive (GU or pelvic) exams with explicit patient permission. |
| Be honest about the realities of the practice, i.e. the challenges. Don't b*tch about it but don't act like a used car salesman. |
| Take a few minutes at the beginning of the shadowing experience to get to know students' background (education level, pre-medicine background, previous EM shadowing, etc). |
| Have the student complete a reflective summary assignment on what they learend. |
| Have goals and objectives that are tied to level of observer. |
| Disseminate shadower expectations in advance - what to expect, what to wear, where to show up, when they will be asked to step out, etc. |
| Establish a clear shadowing system. Use a centralized, accessible process for students to sign up (e.g., calendar, designated contact). Clarify eligibility (e.g., internal vs. external students), any required paperwork or HIPAA training, and share expectations ahead of time—arrival time, attire, and how to find you in the ED. |
| Have a conversation about why they chose to shadow at the beginning of the shift (to set learning objectives). |
| Consider doing bedside rounds as part of the shadow experience. |
| Medical students should receive brief orientation materials about basic expectations and how to best optimize their experience before shadowing in the department. Similarly, shadowing preceptors should receive orientation/ training regarding specific expectations and guidelines when supervising shadowing students. |
| Share what gives you a sense of purpose in and out of the ED and the whys. |
| Communicate with shadowers in advance where to meet you (or how to find you in department). |
| Implement an asynchronous simulation program for education. |
| Have clear rules around patient interactions. |
| Clarify the shadower's goals at the start of the shift - are they there because they want go into EM or are they there for clinical exposure. |
| Be thoughtful about when you schedule shadowers. If feasible, avoid scheduling students during shifts when you're supervising multiple learners or carrying a high clinical burden. |
| Shadowing is "hands-in-pockets" watching and discussing - don't put students in a bad position by offering to let them do hands on care. |
| Have your shadower do a case presentation with a resident mid-shift. |
| Scheduling systems should be in place to avoid overbooking your team/department and taking away from other learners' experiences (balance the number of learners - shadowers, medical students, residents, etc). |
| Don't complain or be derogatory about other staff, specialties, patients, etc. You drag yourself down with them even if there's truth to it. |
| Communicate with shadowers when in the shift you would like them to arrive (i.e. before shift, at start of shift, shortly into shift so that you can get settled/sign-out before shadower, etc). |
| Have a post-shadowing vibe check: would you offer a student the opportunity to do a sub-I, and if so, how will they perform? |
| Schedule the student with preceptors who like their jobs. |
| Shadow times should be 3-4 hours to see the full life span of a patient. |
| Debrief and check in frequently. Critical cases, trauma activations, or unexpected deaths can be intense for learners. If such events occur, check in with the student afterward or plan to debrief during a quieter moment. |
| Be yourself - students want to see our day to day lives. |
| Consider having an ultrasound introduction as part of the shadowing experience. |
| Have shadowers observe resuscitations. |
| Shadowing students should be provided some time during the shadow shift to ask questions or debrief with their preceptor. |
| Limit the total number of learners/trainees to a manageable level. It's a rare faculty who can carry a full clinical team, a student rotator who is getting trained & evaluated, and one or more shadows/preceptees who are just "there for fun", while also looking like a functional attending who is meaningfully contributing to the clinical practice. Something will get sacrificed. Be very cognizant about how and where you are scheduling your shadowers, whether it be yourself, an admin assistant, or the shadowing system that's in place at the department/institution. |
| If it's a particular slow shift with limited learning opportunities, offer for the shadower to come back another day. |
| Have dedicated skill sessions for your shadowers. |
| Have some kind of recognition for your preceptors (monetary or gifts). |
| Some kind of post shadow shift eval (I don't do this regularly, but Ikind of want to). |
| Introduce your student to the team and to patients. Start the shift by introducing your student to other members of the team—residents, nurses, staff, and any other learners. Try to make the student feel welcomed and included. Also be sure to introduce them to patients and briefly explain their role (e.g., “This is Alex, a medical student shadowing with me today”). |
| Shadowing students should have a specific point person assigned as preceptor for each shadowing shift. |
| Be very explicit about whether or not something counts as clinical credit, worth writing a useful LOR for, etc - shadowing very rarely ever is. Communicate that explicitly upfront and early (ideally before they shadow). In fact, if the system your department sets in place can do that for you, even better. |
| If interesting experiences happen in your ED that you are not directly involved with (such as consultant performing a procedure, colleague running a code, nurse placing US guided IV, etc), encourage your student to watch that experience. |
| Have your shadow program be a separate paid process. |
| If your shadowing program is part of official program, ensure there is credit for students. |
| Involve the student when appropriate for their level, following hospital/medical school policies. For example, an M3 student can likely be more actively involved than an early M1. |
| Normalize questions and curiosity, while explaining that there may be times when you need to defer questions to a less busy moment. |
| Have your shadower participate in didactics. |
| Have your shadower complete a shift with your nursing staff. |
| Medical students can shadow senior residents who have had appropriate training/orientation to a shadowing program. |
| Ideally, have a department level shadowing system in place with appropriate administrative support, rules, ways to process/schedule students in a way that's compliant with hospital policy, a way to keep track of who's shadowing who and to give credit to those doing the work, etc. |
| If you are going to be documenting for long periods of time and need to focus, try to find other ways to keep shadower engaged such as following one of your colleagues/residents/attendings/senior medical students or to pair up with a nurse/tech for a short period of time (could even have them watch triage). |
| Students that are shadowing can observe but should not participate in or perform patient care encounters and procedures unless specific types of encounters or procedures are clearly defined by the program/institution and appropriately supervised by the preceptor. |
| Have a limitation on who can observe! We get SO MANY international requests from random people. Better to have some connection to the department or school. |
| Have the shadower complete some sort of output such as a journal or case report. |
| Each student should have a designated preceptor for the shift. However, students can spend time with another member of the team—such as a resident, nurse, or another attending—if a unique learning opportunity arises. |
| Students that are shadowing should not be expected to take on "scut" or other noneducational tasks. |
